# Disparities in the use of mobile phone for seeking childbirth services among women in the urban areas: Bangladesh Urban Health Survey

**DOI:** 10.1186/s12911-017-0578-2

**Published:** 2017-12-29

**Authors:** Ghose Bishwajit, Md. Rakibul Hoque, Sanni Yaya

**Affiliations:** 1 Institute of Nutrition and Food Science, University of Dhaka, Dhaka, 1000 Bangladesh; 2 Faculty of Business Studies, Department of Management Information Systems (MIS), University of Dhaka, Dhaka, 1000 Bangladesh; 3 Faculty of Social Sciences, School of International Development and Global Studies, University of Ottawa, Ottawa, Canada

**Keywords:** mHealth, Neighbourhood disparity, Reproductive health service, Bangladesh Urban Health Survey

## Abstract

**Background:**

In Bangladesh, similar to its other South Asian counterparts, shortage of health workers along with inadequate infrastructure constitute some of the major obstacles for the equitable provision of reproductive healthcare services, particularly among the marginalized and underserved neighbourhoods. However, given the rapidly expanding broadband communication and mobile phone market in the country, the application of eHealth and mHealth technologies offer a window of opportunities to minimise the impact of socioeconomic barriers and promote the utilization of maternal healthcare services thereby. In the present study we aimed to investigate 1) the prevalence of usage of mobile phones for seeking childbirth services, 2) neighbourhood and socioeconomic disparities in the use, and 3) association between using mobile phones and the uptake of postnatal care among mothers and neonates.

**Methods:**

Data for the present study came from Bangladesh Urban Health Survey 2013. Study subjects were 9014 married women aged between 15 and 49 years.

**Results:**

The overall rate of use of mobile phone was highest in City Corporation non-Slum areas (16.2%) and lowest in City Corporation Slum areas (7.4%). The odds of using mobile for seeking childbirth services were significantly higher among those who were living in non-slum areas, and lower among those who never attended school and lived in poorer households. Results also indicated that women in the slum areas who used mobile phone for childbirth service seeking, were 4.3 times [OR = 4.250;95% CI = 1.856–9.734] more likely to receive postnatal care for themselves, and those from outside the city-corporation areas were 2.7 times [OR = 2.707;95% CI = 1.712–4.279] more likely to receive postnatal care for the newborn.

**Conclusion:**

Neighbourhood, educational and economic factors were significantly associated with the mobile phone utilization status among urban women. Promoting access to better education and sustainable income earning should be regarded as an integral part to the expansion of mHealth for maternal healthcare seeking behaviour.

## Background

Historically, the demography of Bangladesh has been characterized by high fertility and widespread prevalence of infant and maternal mortality ratio (MMR). Thanks to the concrete policy-oriented actions within the framework of the millennium development goals (MDG-5A), the country has experienced remarkable progress in reducing MMR during last two decades. Between 1998 and 2010, MMR fell from 322 to 194/100,000 livebirths with an average annual decline of 5·6% [1, 2]. According to the analysis of a recent study based on Bangladesh Maternal Mortality Surveys and Demographic and Health Surveys, this progress is attributable to improved access to reproductive health services together with falling fertility and poverty and rising literacy rates, and thus averted an estimated 52% of maternal deaths during the period [1]. However, the provision of universal access to reproductive healthcare (MDG-5B) and the utilization of maternal healthcare services (MHS) are far behind the internationally agreed-upon targets [3, 4]. A major barrier for meeting this target is an acute human resource crisis in the healthcare system in Bangladesh [3, 5].

Despite many attempt to increase the supply of skilled health workers in recent years, Bangladesh continues to be one of the 57 countries with a serious shortage of trained doctors, paramedics, nurses and midwives [6, 7], with a meagre three physicians and two nurses per 10,000 population in 2008 [7]. The crisis gets even more aggravated given the highly skewed distribution of the qualified service providers between urban and rural areas as about 86% of the physicians; and 75% of the nurses and dentists are based in urban areas where less than a quarter of the total population lives [7]. Given the current level of production of trained health workforce, it is very unlikely that the situation will change at required scale in the near future.

Under these circumstances, the use of Information and Communication Technology (ICT), e.g. mobile phone connectivity can be regarded as a novel and cost-effective way to tackle the current health worker crisis. Therefore, the prospect of eHealth and mHealth technology to circumvent the persistent problems in healthcare (such as governance, transparency, infrastructure, patient empowerment) needs to be assessed. This is particularly important to understand the factors that facilitate or limit the usage of technology for healthcare services at population level. A growing number of researches are being dedicated to explore people’s attitude towards using technologies for healthcare. For instance, the impact of smart phone applications, social media sites for sharing and learning health related knowledge have been studied extensively [8–11]. However, similar research evidences are limited for South Asian countries like Bangladesh, especially in the areas of maternal healthcare utilization. Therefore, we conducted this study to explore the patterns of mobile health use for childbirth related services. Since surveys on this topic are hard to organize, we used secondary datasets from Bangladesh Urban health survey, which was conducted in 2013. The survey was designed to examine the health and service utilization profile of the urban population with a special focus to examine the disparity between slum and non-slum dwellers [12]. This was the first survey in Bangladesh to include subjects from the urban areas including City Corporation: Slum, City Corporation: non-Slum, Rest-Urban (e.g. suburb areas) from all seven districts that provided information on mobile phone usage for seeking childbirth services. The information collected in the 2013 UHS do not provide precise details about the types of services sought, and whether the service was helpful. However, the information contained in it can be instrumental in designing more comprehensive studies in future and give directions for urban health programs in the country.

## Methods

### Data collection

The dataset used in this study was obtained from Bangladesh Urban Health Survey (BUHS) 2013. BUHS is a representative household survey of slums and non-slums of City Corporations and other urban areas. The survey was undertaken as a response to the growing concern regarding the fast urbanization, and associated transitions in demography, socioeconomic and population health sectors. The survey was made possible by the collaborative effort of the National Institute of Population Research and Training (NIPORT), Measure Evaluation, University of North Carolina at Chapel Hill, USA, and icddr,b [12]. Field work was carried out by The Associates for Community and Population Research with financial assistance by the United States Agency for International Development (USAID) and the United Kingdom’s Department for International Development (DFID). More details regarding the objectives and sampling procedures were published elsewhere [12].

### Variables

The outcome variable was mobile phone utilization status for seeking childbirth services. This included services availed though mobile phones from professional (physicians, nurses); informal relational (parents, friends, teachers) and informal personal (self-medication/browsing Internet) sources [13]. Participants were asked whether or not they used mobile phone during last pregnancy/delivery to get services. The responses were categorized as ‘Yes’ and ‘No’.

The main explanatory variables were type of neighbourhood, evaluated by the area of residence divided into: City Corporation: Slum, City Corporation: non-Slum, Rest-Urban. And socioeconomic status measure by attendance of school and household wealth status. Wealth status was calculated based on household characteristics (access to electricity, source of drinking water, type of toilet facilities, household construction material), ownership of durable goods, and the household. Each item was assigned a factor score and the weighted sum was calculated to form the wealth index. The index then was divided into quintiles and categorized as lowest to highest, with higher quintiles indicating higher wealth status.

Based on literature review, several other variables were selected in the analysis as potential confounders: Age (14–19/20–24/25–29/30–34/34+), Religion (Islam/Hinduism/Buddhism), Ever attended school (Yes/No), Wealth quintile (Poorest/Poorer/Middle/Richer/Richest), Currently working (Yes/No), Parity(1, 2, 3, >3), Mother’s club membership(Yes, No), Microcredit borrower (No/Yes), Watches TV (Yes/No), Listens to radio (Yes/No).

### Analytical procedure

Data analyses were carried out by using R statistical software. The dataset was checked for cases which fulfilled all the inclusion criteria: having experienced at least one childbirth in the past 5 years, availability of information on mobile phone usage (usage refers to seeking service for childbirth purposes only throughout the manuscript). To ensure data accuracy, the new dataset was checked for outliers and tested multicollinearity issues. In total 9014 cases were retained in the final dataset. Participants’ characteristics were presented using percentages. Contingency table was used and chi-square test was performed to examine the bivariate tests of association between mobile phone usage and the explanatory variables. Following bivariate analysis, multivariable regression analysis was used to calculate the odds ratios of using mobile phones with corresponding 95%CI. The explanatory variables that were significant at <0.25 were included in the regression model [14]. We additionally checked for the multivariable association between using mobile phone and receiving postnatal care for participants and the newborn. For multivariable analyses, *p*-values (two-tailed) were considered statistically significant only when below 0.05.

## Results

Basic sample characteristics were shown in Table 1. Mean age of the participants was 25.92 (+/−5.6) years. Results show that majority of the participants were living in the slum areas (45.1%), and less than a quarter in the City Corporation areas. Most of the women were 20–24 years of age (34.7%), belonged to Islam faith (93.9%), and reported ever attending school (84.1%). Little less than one-third (30.9%) were living in the poorest households and 10.9% were from richest households. Only 17.3% had an employment at the time of the survey. More than two-fifth were primiparous (41%). Only 3% were members of mothers club, and 13.9% were borrowers of microcredit. Rate of TV watching was high (87.1%) compared to that of listening to radio (3.7%). Rate of use of mobile phone in City Corporation Slum, City Corporation non-Slum and Rest-Urban areas were respectively 7.4, 16.2, and 12.7%.

**Table 1:** Basic demographic and socioeconomic characteristics of the participants

Variables	Total	City Corporation: Slum (*n* = 4066, 45.1%)	City Corporation: non-Slum (*n* = 2076, 23%)	Rest-Urban (*n* = 2872, 31.9%)
Age	(25.92+/−5.6)			
14–19	924 (10.3)	11.4	7.8	10.5
20–24	3124(34.7)	36.7	31.8	33.8
25–29	2736 (30.4)	28.8	33.0	30.7
30–34	1482 (16.4)	14.9	19.1	16.7
34+	748 (8.3)	8.2	8.3	8.4
Religion				
Islam	8465 (93.9)	94.2	94.7	92.9
Hinduism	514 (5.7)	5.6	4.8	6.5
Buddhism	35 (0.4)	0.2	0.4	0.6
Ever attended school				
Yes	7583 (84.1)	76.1	92.5	89.5
No	1431 (15.9)	23.9	7.5	10.5
Wealth quintile				
Poorest	2788 (30.9)	43.6	8.8	29.1
Poorer	2187 (24.3)	30.9	15.2	21.4
Middle	1752 (19.4)	18.4	19.8	20.6
Richer	1301 (14.4)	5.6	24.7	19.5
Richest	986 (10.9)	1.5	31.5	9.4
Currently working				
Yes	1562 (17.3)	24.9	12.6	10.0
No	7452 (82.7)	75.1	87.4	90.0
Parity				
1	3694 (41.0)	39.6	42.7	41.7
2	2923 (32.4)	29.8	35.6	33.8
3	1445 (16.0)	17.1	14.8	15.4
> 3	952 (10.6)	13.5	6.8	9.1
Mother’s club				
Yes	270 (3.0)	3.2	1.8	3.6
No	8744 (97.0)	96.8	98.2	96.4
Microcredit				
No	7761 (86.1)	87.0	92.2	80.4
Yes	1253 (13.9)	13.0	7.8	19.6
Watches TV				
Yes	7847 (87.1)	84.2	93.9	86.2
No	1167 (12.9)	15.8	6.1	13.8
Listens to radio				
Yes	333 (3.7)	3.2	5.7	2.9
No	8681 (96.3)	96.8	94.3	97.1
Used mobile phone				
Yes	1001 (11.1)	7.4	16.2	12.7
No	8013 (88.9)	92.6	83.8	87.3

Rate of participation from seven districts were respectively 2.3, 22.5, 55.8, 6.3, 5.9, 4.0, and 3.1% (Result not shown).

### Cross-tabulation

Results of chi-square tests of association (Table 2) indicated that two-thirds of the women who reported using mobile phone for delivery-care service were of 20–24 years of age, more likely to be followers of Islam faith (Rest-urban), ever attended school, were living in the richest households, were unemployed, had two children, did not have mother club and microcredit membership, watched TV and did not listen to radio. However, the association varied substantially after stratification into slum and non-slum areas. In the stratified analysis, only ever attended school, household wealth status, and TV watching status appeared to be signification for three types of neighbourhoods.

**Table 2:** Bivariate association between mobile utilization for seeking childbirth services with the socioeconomic variables stratified by neighbourhood types

Variables	n (%)	City Corporation: Slum (7.4%)	City Corporation: non-Slum (16.2%)	Rest-Urban (12.2%)
Age				
14–19	88 (8.8)	9.3	8.6	8.5
20–24	339 (33.9)	38.5	27.7	35.7
25–29	332 (33.2)	29.9	33.6	35.4
30–34	177 (17.7)	16.3	22.9	14.0
34+	65 (6.5)	6.0	7.1	6.3
p	.027	.397	.171	.064
Religion				
Islam	925 (92.4)	93.7	95.2	88.7
Hinduism	72 (7.2)	6.0	4.8	10.4
Buddhism	4 (0.4)	0.3		0.8
p	.097	.817	.417	.004
Ever attended school				
Yes	935 (93.4)	84.4	97.6	97.0
No	66 (6.6)	15.6	2.4	3.0
p	<.0001	<.0001	<.0001	<.0001
Wealth quintile				
Poorest	142 (14.2)	29.6	2.1	12.6
Poorer	200 (20.0)	35.5	10.1	16.2
Middle	190 (19.0)	23.9	13.7	19.8
Richer	226 (22.6)	9.0	25.3	31.3
Richest	243 (24.3)	2.0	48.8	20.1
p	<.0001	<.0001	<.0001	<.0001
Currently working				
Yes	133 (13.3)	17.9	13.7	9.1
No	868 (86.7)	82.1	86.3	90.9
p	<.0001	.004	.529	.575
Parity				
1	473 (47.3)	43.9	47.6	49.7
2	334 (33.4)	32.9	35.1	32.1
3	140 (14.0)	15.0	12.8	14.3
4	54 (5.4)	8.3	4.5	3.8
p	<.0001	.019	.08	<.0001
Mother’s club				
Yes	24 (2.4)	2.0	2.4	2.7
No	977 (97.6)	98.0	97.6	97.3
	.139	.304	.378	.45
Microcredit				
No	869 (86.8)	85.7	91.1	83.8
Yes	132 (13.2)	14.3	8.9	16.2
p	.262	.272	.23	.047
Watches TV				
Yes	944 (94.3)	91.0	97.6	94.0
No	57 (5.7)	9.0	2.4	6.0
p	<.0001	<.0001	.001	<.0001
Listens to radio				
Yes	66 (6.6)	5.0	10.7	4.1
No	935 (93.4)	95.0	89.3	95.9
p	<.0001	.055	<.0001	.103

BUHS 2013

### Multivariable regression analysis

Table 3 shows that in the fully adjusted model, the odds of using mobile for seeking childbirth services were 28% higher [OR = 1.282;95% CI = 1.076–1.526] among those who were living in non-slum areas and 36% [OR = 0.639;95% CI = 0.486–0.839] lower among those who never attended school. Results also indicated that compared with women who lived in the poorest households, those who lived in the poorer, middle, richer and richest wealth status households had respectively 46% [OR = 1.464; 95% CI = 1.186–1.807], 2.3 times [OR = 2.279; 95% CI = 1.820–2.855], 2.5 times [OR = 2.512; 95% CI = 1.989–3.173] and 4.1 times [OR = 4.108;95% CI = 3.145–5.367] higher odds of mobile phone use for childbirth service seeking.

**Table 3:** Multivariable analysis on the association between mobile phone use for childbirth service seeking and neighborhood and socioeconomic characteristics

	Model 1 (OR, 95% CI)	Model 2 (OR, 95% CI)	Model 3 (OR, 95% CI)
Type of neighborhood			
City Corporation: Slum	Ref	Ref	Ref
City Corporation: Non-slum	1.815 (1.546–2.132)	1.263 (1.061–1.504)	1.282 (1.076–1.526)
Rest Urban	0.752 (0.640–0.882)	1.074 (0.902–1.278)	1.085 (0.911–1.292)
Ever attended school			
Yes	Ref	Ref	Ref
No	0.344 (0.266–0.444)	0.621 (0.473–0.815)	0.639 (0.486–0.839)
Wealth quintile			
Poorest	Ref	Ref	Ref
Poorer	1.556 (1.269–1.907)	1.496 (1.213–1.845)	1.464 (1.186–1.807)
Middle	2.689 (2.182–3.313)	2.348 (1.876–2.938)	2.279 (1.820–2.855)
Richer	3.249 (2.646–3.989)	2.645 (2.098–3.335)	2.512 (1.989–3.173)
Richest	6.094 (4.879–7.612)	4.597 (3.553–5.947)	4.108 (3.145–5.367)

BUHS 2013

N.B. Model 1 = Univariate model. Model 2 = adjusted for Age, Religion, Attending school, Wealth quintile, Currently working, Parity; Model 3 = Age, religion, attending school, wealth quintile, Currently working, Parity, Watches TV, Listens to radio

Table 4 highlights that women in the slum areas who used mobile phone for childbirth service seeking, were 4.3 times [OR = 4.250;95% CI = 1.856–9.734] more likely to receive postnatal care for themselves, and those from outside the city-corporation areas were 2.7 times [OR = 2.707;95% CI = 1.712–4.279] more likely to receive postnatal care for the newborn. No significant association was observed for women in the City Corporation: non-slum areas.

**Table 4:** Odds of availing postnatal care among respondents and for newborns according to mobile phone usage status

Used mobile phone for delivery services	City Corporation: Slum (OR, 95% CI)	City Corporation: non-Slum (OR, 95% CI)	Rest-Urban (OR, 95% CI)
Postnatal care for respondents			
No	Ref	Ref	Ref
Yes	4.250 (1.856–9.734)	1.448 (0.726–2.888)	0.650 (0.406–1.039)
Postnatal care for newborns			
No	Ref	Ref	Ref
Yes	1.069 (0.784–1.456)	0.860 (0.637–1.161)	2.707 (1.712–4.279)

N.B. Adjusted for Age, region, religion, attending school, wealth quintile, currently working, Parity, Watches TV, Listens to radio

## Discussion

Based on datasets from the second wave of the Bangladesh Urban Health Survey, the present study aimed to explore the patterns of mobile phones usage for seeking delivery care services. The survey was originally designed to monitor various health service utilization among the burgeoning urban population in the country. Overall prevalence of mobile phone usage was about one in ten women with the highest rate of usage of in City Corporation non-slum and lowest in City Corporation slum areas. The comparatively lower usage of mobile phone in the slum areas can be explained from two perspectives. Firstly, women from lower socioeconomic neighbourhoods are usually less likely to deliver at a health facility [15, 16]. Secondly, poor health/eHealth literacy can limit the usage of phone for health service seeking purposes [17]. Apart from the usage of mobile phones, important disparities were observed in educational and socioeconomic terms. The rate of ever attending school was lowest, and the percentage of living in the poorest and poorer was highest among women living in the slum areas. Poor health literacy and lower socioeconomic status act as limiting factors to accessing and proper utilization of healthcare resources and opportunities available to them. Therefore, programs aimed at promoting reproductive health services should take these factors into account for effective expansion of general and eHealth service utilization. This findings are therefore of significant importance for urban slum area development, and for health promotion programs for the slum dwellers as well.

Regional differences were also apparent as the usage of mobile phone among slum dwellers varied from about 65% in Dhaka to as low as 0.7% in Rajshahi. Moreover, exacerbating livelihood insecurity among the agrarian communities owing to rising intensity and frequency of natural disasters (such as flood and seasonal drought in the northern region of the country) has made the rate of urbanization uncontrollably fast [18]. This is a serious concern for the already overburdened healthcare system as the demand for service far exceeds the current capacity of public health facilities. Given these circumstances, the adoption and advancement of eHealth technologies, and supply of eHealth professionals need to be regarded as an urgent imperative. With a booming domestic cellular market and constantly improving network and interest coverage, mobile phones certainly hold a promising future for the healthcare system in Bangladesh.

As expected, results from the regression analyses highlighted a strong association between usage of mobile phone with types of neighbourhood, educational and household wealth status. As discussed above, addressing the barriers to education and sustainable income earning should be regarded as an integral part to promoting eHealth, and overall healthcare seeking behaviour among women. Findings also indicate that participants who used mobile phones were more likely to receive postnatal services for themselves in the slum areas, and for their babies in the Rest-urban areas. Post-natal checkup are considered crucial to prevent infant mortality [16, 19], and to reduce childbirth related complication among mothers [20]. Thus, although the rate of usage of mobile phones is currently low, it can actually offer potential opportunities to promote the uptake of postnatal services for mother and infants especially in the disadvantaged urban neighbourhoods.

Although being a predominantly rural population (64.97% as per 2016 World Bank estimate) with a high proportion living in off-grid areas, the rate of computer and mobile phone ownership and internet use has been rising steadily especially in the rural areas [21].

According to the reports by Household Income and Expenditure Survey (HIES), the percentage of mobile phone ownership in the rural areas rose from 6.05% in 2005 to 56.77% in 2010, compared to from 26.73 to 82.74% in the urban areas during the same period [22]. At national level, the coverage increased from 11.29% to more than 63% [22].

This study is first to report the usage of mobile phones for seeking childbirth services among women in Bangladesh. One particular strength of the survey was its generalisability for urban women as subjects were recruited from all seven districts in the country. Currently there is a scarcity of population based researches on usage of mobile phones for health care purposes in the country. Therefore, the present study is expected to provide the incentives for further explorations on the barriers to the adoption of this technology at population level. More importantly, the coverage of reproductive health services is shockingly poor in terms of extent and quality of the service provided. Hence, understanding the usability of mobile phones especially for maternal health services is particularly essential.

Apart from the important contributions, the limitations of this study also need to be laid out and taken into consideration when interpreting the results. Firstly, there was no precise information on the usage of mobile phones, and thus it was not possible to know if the communication took place with professional service providers. In Bangladesh, a large proportion of children are born at home and without the assistance of skilled birth attendant (SBA). Therefore, there was no way to determine if the communication finally resulted in uptake of the service, and/or service was sought from the proper provider. Secondly, women who deliver at a health facility are generally more likely to get themselves and their babies screened for post-delivery complications [23, 24], regardless of using or being able to get service through mobile phone communication. As the information were self-reported, and not measured objectively, there were chances for reporting bias or error that might have influenced the associations. It is also possible that the use of mobile phones for childbirth seeking was more fashionable during the survey year compared to the previous (five) years, which might have impacted the prevalence of usage. Studies have shown an upward trend in seeking healthcare or medication related information through mobile phone/internet in both developed and developing countries [25, 26]. Last but not least, the data were cross-sectional and provides no indication of causality in the association between the variables.

## Conclusions

This study concludes that rate of utilization of mobile phone to seek childbirth related services among urban women was low, with noticeable variation among slum and non-slum dwellers. Besides the types of neighbourhood, educational and household wealth status also appeared to be significantly associated with the utilization status of mobile phones. Another important observation was that, those who reported using mobile phones for childbirth related services in slum areas were more likely to receive postnatal care for themselves, and for their babies in the rest-urban areas. Addressing the neighbourhood and socioeconomic factors that barrier the utilization of mobile phones may prove beneficial for the sustainable expansion of eHealth and mHealth services for reproductive care among urban women. Further studies are recommended to focus on the precise purposes for and efficacy of mobile phone usage for maternal healthcare service seeking include antenatal and postnatal care services.

## Abbreviations

BUHS: Bangladesh Urban Health Survey
ICT: Information and Communication Technology
MDG-5A: Millennium development goals
MHS: Maternal healthcare services
SBA: Skilled birth attendant
